# Granulocyte-Colony Stimulating Factor-Induced Neutrophil Recruitment Provides Opioid-Mediated Endogenous Anti-nociception in Female Mice With Oral Squamous Cell Carcinoma

**DOI:** 10.3389/fnmol.2019.00217

**Published:** 2019-09-16

**Authors:** Nicole N. Scheff, Robel G. Alemu, Richard Klares, Ian M. Wall, Stephen C. Yang, John C. Dolan, Brian L. Schmidt

**Affiliations:** ^1^Bluestone Center for Clinical Research, New York University, New York, NY, United States; ^2^College of Dentistry, New York University, New York, NY, United States

**Keywords:** pain, neutrophils, anti-nociception, opioids, squamous cell carcinoma, sex differences, cancer

## Abstract

Oral cancer patients report severe function-induced pain; severity is greater in females. We hypothesize that a neutrophil-mediated endogenous analgesic mechanism is responsible for sex differences in nociception secondary to oral squamous cell carcinoma (SCC). Neutrophils isolated from the cancer-induced inflammatory microenvironment contain β-endorphin protein and are identified by the Ly6G^+^ immune marker. We previously demonstrated that male mice with carcinogen-induced oral SCC exhibit less nociceptive behavior and a higher concentration of neutrophils in the cancer microenvironment compared to female mice with oral SCC. Oral cancer cells secrete granulocyte colony stimulating factor (G-CSF), a growth factor that recruits neutrophils from bone marrow to the cancer microenvironment. We found that recombinant G-CSF (rG-CSF, 5 μg/mouse, intraperitoneal) significantly increased circulating Ly6G^+^ neutrophils in the blood of male and female mice within 24 h of administration. In an oral cancer supernatant mouse model, rG-CSF treatment increased cancer-recruited Ly6G^+^ neutrophil infiltration and abolished orofacial nociceptive behavior evoked in response to oral cancer supernatant in both male and female mice. Local naloxone treatment restored the cancer mediator-induced nociceptive behavior. We infer that rG-CSF-induced Ly6G^+^ neutrophils drive an endogenous analgesic mechanism. We then evaluated the efficacy of chronic rG-CSF administration to attenuate oral cancer-induced nociception using a tongue xenograft cancer model with the HSC-3 human oral cancer cell line. Saline-treated male mice with HSC-3 tumors exhibited less oral cancer-induced nociceptive behavior and had more β-endorphin protein in the cancer microenvironment than saline-treated female mice with HSC-3 tumors. Chronic rG-CSF treatment (2.5 μg/mouse, every 72 h) increased the HSC-3 recruited Ly6G^+^ neutrophils, increased β-endorphin protein content in the tongue and attenuated nociceptive behavior in female mice with HSC-3 tumors. From these data, we conclude that neutrophil-mediated endogenous opioids warrant further investigation as a potential strategy for oral cancer pain treatment.

## Introduction

Oral cancer patients report severe function-induced pain; patients experience impaired speech, swallowing, eating, and drinking (Bjordal et al., 2001) and severity is greater in females. We previously demonstrated a sex difference in the prevalence and severity of oral squamous cell carcinoma (SCC)-induced nociception (Scheff et al., 2018). Female mice with 4-nitroquinoline-1-oxide (4NQO)-induced oral SCC exhibited more orofacial nociceptive behavior compared to male mice (Scheff et al., 2018). Furthermore, infiltrating neutrophils contribute to decreased nociceptive behavior in male mice during early 4NQO-induced carcinogenesis through opioid-mediated endogenous anti-nociception (Scheff et al., 2018). Activation of opioid receptors on peripheral sensory nerves can produce anti-nociception (Stein et al., 1991, 2003; Mousa et al., 2007; Hua and Cabot, 2010). Clinical and preclinical evidence suggest that endogenous opioids can be released in local inflamed tissues to alleviate inflammatory hyperalgesia (Kapitzke et al., 2005; Iwaszkiewicz et al., 2013). A major source of opioid peptides in peripheral tissues is non-neuronal cells (Kapitzke et al., 2005); keratinocytes and immune cells contain and release met-enkephalin and β-endorphin (Stein et al., 1990; Khodorova et al., 2003; Smith, 2003; Slominski et al., 2011). Peripheral immune-mediated opioid anti-nociception is restricted to the inflammatory site without side effects in response to opioid receptor activation in the central nervous system (Kapitzke et al., 2005). We hypothesize that neutrophil recruitment could be exploited as a therapeutic approach to alleviate oral cancer pain in female mice.

In the early stage of inflammation, opioid-producing neutrophils comprise the majority of infiltrating immune cells (Rittner et al., 2001; Brack et al., 2004b). Oral cancer cells secrete hematopoietic growth factor granulocyte colony stimulating factor (G-CSF; Hayashi et al., 1995; Lee et al., 2013), which results in neutrophil infiltration into the cancer (Demetri and Griffin, 1991). Administration of recombinant G-CSF (rG-CSF) has been used clinically to increase the neutrophil count when treating chemotherapy- or radiotherapy-induced neutropenia (Dale, 2002; Lambertini et al., 2014). rG-CSF-generated neutrophils have the potential to secrete opioids and subsequently reduce nociceptive signaling. Chao et al. (2012) demonstrated that rG-CSF administration in rats with chronic nerve constriction injury alleviated mechanical allodynia and thermal hyperalgesia. rG-CSF-mediated increase in the recruitment of opioid-containing neutrophils was confirmed as the source for the anti-nociception (Chao et al., 2012).

To investigate whether rG-CSF increased neutrophil infiltration to the oral cancer microenvironment and alleviated oral cancer pain, we used two oral cancer pain mouse models: (1) an oral cancer pain model created by injecting supernatant from human oral cancer cell lines into the tongue; and (2) a human tongue xenograft cancer model created by injecting oral cancer cells into the tongue. The dolognawmeter assays (Dolan et al., 2010) were used to quantify a behavioral index (gnawing) of orofacial nociception in both models. rG-CSF treatment increased Ly6G^+^ neutrophil recruitment to the oral cancer microenvironment and reduced oral cancer-induced nociception in an endogenous opioid-mediated analgesic mechanism in a sex-dependent manner.

## Materials and Methods

### Animals

Male and female adult (10–12 weeks, 20–30 g) C57BL/6 mice (Jackson Labs, Bar Harbor, ME, USA) were used for oral cancer supernatant experiments. The xenograft cancer model required adult nude athymic mice (Jackson Labs, Bar Harbor, ME, USA). Mice were maintained on a 12:12 h light cycle and were housed in temperature-controlled rooms with access to food and water. Researchers were trained under the Animal Welfare Assurance Program. Experimental procedures were approved by the New York University Institutional Animal Care and Use Committee and were conducted in line with the National Institutes of Health guidelines for the use of laboratory animals in research.

### Cell Culture and Supernatant Collection

Oral cancer cell line, HSC-3 (JCRB, Sekisui Xenotech, Kansas City, KS, USA), was cultured in 10 cm^2^ cell culture dishes at 37°C with 5% CO_2_ in Dulbecco’s modified Eagle’s medium (DMEM, Gibco, Waltham, MA, USA) supplemented with 10% fetal bovine serum and penicillin/streptomycin (50 U/mL). For collection of supernatant, the culture medium was changed to serum-free DMEM without phenol red (3 mL total volume) when cells reached 70%–80% confluency (1.5 × 10^6^ cells) and cells were subsequently incubated for an additional 48 h. Cell culture supernatant was collected, centrifuged at 300× *g* to remove cell debris, and frozen at −20°C until needed. HSC-3 cell culture supernatant was collected from passage 8.

### Dolognawmeter Behavior Assay

Dolognawmeters were used in parallel to quantify a behavioral index (gnawing activity) of orofacial nociception in mice (Dolan et al., 2010). Each mouse was placed in a cylindrical confinement tube. Two polymer dowels in series prevent the mouse from progressing forward in the tube. To escape the tube, the mice gnaw through the two dowels. Each dowel is connected to an electronic timer. The timers record the duration of gnawing required to sever the dowels. The outcome variable is the time required (gnaw-time) to sever the second dowel. Prior to the experimental trials, mice were trained for 10 sessions to acclimatize the animals to the dolognawmeter and to establish a baseline gnaw-time (the mean of the last three gnawing trials). Once the baseline gnaw-time measurements were established, drug/treatment injections were administered, followed by behavioral testing.

### Conditioned Place Preference Assay

Conditioned place preference (CPP) to pain relief has been previously used to reveal underlying mechanisms of ongoing pain in several models including oral cancer pain (King et al., 2009; Chodroff et al., 2016). We determined whether synthetic met-enkephalin analog, DAMGO (3 μg/kg i.p.), produces CPP in mice with HSC-3 tongue xenografts. We performed a single trial CPP protocol on post-inoculation day (PID) 21 through 25. The 3-chamber CPP apparatus consists of two conditioning chambers with distinct tactile, visual, and olfactory cues, connected by a smaller neutral chamber that was brightly lit. The visual cues were horizontal stripe and dot wall papers. The tactile cues were smooth and rough flooring. The olfactory cues were strawberry and mint. White noise was played to provide background noise and block out any extraneous sounds. On the first day (PID 21, preconditioning) of the experiment, mice were introduced to the neutral chamber and allowed to explore all three chambers for 1 h. Baseline time spent in the chambers was measured using ANY-maze tracking software (Braintree Scientific, Braintree, MA, USA). Exclusion criteria included mice were spending <20% or >80% time in a chamber. Mice were assigned treatment-chamber pairings using a counterbalanced design for the following three consecutive days. On the second, third and fourth days (PID 22–24, conditioning), mice received i.p. injection of saline followed by confinement into the appropriate pairing chamber for 1 h, following which they were returned to their home cage. Four hours later, mice received i.p. injection of DAMGO (3 μg/kg, 50 μl) followed by confinement into the opposite pairing chamber for 1 h. On the fifth day (PID 25, testing), mice were once again allowed to freely explore the apparatus for 1 h. Time spent in each chamber was recorded by ANY-maze. The experimenter conducting the behavioral tests (IW) was blinded to the treatment groups.

### Acute Supernatant Oral Cancer Pain Model

We developed a model of acute oral cancer pain by injecting cancer cell line supernatant into the tongues of mice. Under isoflurane general anesthesia, 50 μl injections of oral cancer (HSC-3) supernatant was administered into the anterior lateral tongue over a 5 s period. A 5 μg dose of rG-CSF was injected intraperitoneal (i.p.) 24 h prior to the supernatant injection to increase neutrophil infiltration in the tongue. The dose and route of administration for rG-CSF treatment was determined in a pilot study using 0.2 μg/mouse (low dose) and 5 μg/mouse (high dose) of rG-CSF and two different routes of administration: subcutaneous into the tongue (s.c.) and intraperitoneal (i.p.). An index of cancer-induced nociception was quantified with the dolognawmeter assay. Naloxone (500 μg/kg; Sigma Aldrich, St. Louis, MO, USA) was co-injected with HSC-3 cell culture supernatant for experiments designed to inhibit endogenous opioid-mediated analgesic signaling in response to rG-CSF treatment and oral cancer supernatant injection in female and male mice. Neutrophil infiltration in the tongue was measured with flow cytometry 24 h after the supernatant injection. The experimenter conducting the behavioral tests (RA) was blinded to the treatment groups.

### Xenograft Oral Cancer Pain Model

We used the human tongue xenograft cancer mouse model to determine whether chronic rG-CSF treatment decreases oral cancer-induced nociception. Mice were inoculated with 1 × 10^5^ HSC-3 cells in 30 μL of 1:1 DMEM and Matrigel into the anterior lateral portion of the tongue as previously described (Ye et al., 2018). Nociceptive behavior was measured twice per week using a dolognawmeter assay for the duration of the experiment. A 2.5 μg dose of rG-CSF was injected (i.p.) 24 h prior to the nociceptive behavior assessment to increase neutrophil infiltration in the tongue. Body weight was recorded once per week. Tongue tumor size was quantified at PID 38. The tongue was fixed in 10% neutral buffered formalin, bisected, paraffin-embedded with cut side down, and sectioned at 5 μm thickness through the entire block (about 50 sections). Average tumor area relative to total tongue area in the 1st, 10th, 20th, 30th and 40th section was quantified using hematoxylin and eosin (H&E) stain and ImageJ (NIH, Bethesda, MD, USA). The experimenters conducting the behavioral tests (RA) and tumor quantification (RA, RK) were blinded to the treatment groups.

### Tongue Tissue Dissociation

Mouse tongues were harvested and dissociated as previously described (Scheff et al., 2018). Briefly, tongue tissue was dissected and minced in DMEM with antibiotics, collagenase-H (0.5 mg/mL; Sigma Aldrich, 34 units/mg), DNase (0.5 mg/mL) and 20 mM 4-(2-hydroxyethyl)-1-piperazine ethanesulfonic acid (HEPES), and then incubated at 37°C for 1 h. The tissue was then mechanically dissociated using a fire-polished pipette, washed twice with fresh DMEM containing antibiotics and HEPES, and resuspended in Ca^2+^/Mg^2+^ free phosphate buffered saline (Sigma Aldrich) containing 3% fetal bovine serum, 1 mM EDTA, and 0.02% sodium azide and filtered through a 40 μm cell strainer (Falcon brand, Fisher Scientific, Waltham, MA, USA).

### Flow Cytometry

Flow cytometry was used to quantify immune cell subtypes in tongue tissue from female and male mice. The antibody panel and flow cytometry gating strategy were used as previously defined (Scheff et al., 2017). Within CD45^+^ hematopoietic cells, neutrophils and monocytes/macrophage were detected and quantified using antibodies specific to receptors expressed on each cell type. Single-cell suspensions were prepared and samples were incubated in rat anti-mouse purified CD16/CD32 to block nonspecific FC receptor binding. CD45 monoclonal antibody (mAb) conjugated with V450 dye (1:400; BD Biosciences Franklin Lakes, NJ, USA) was used to label all hematopoietic cells. To differentiate leukocyte subpopulations, we stained cell suspensions with fluorescently conjugated rat anti-mouse mAbs recognizing neutrophils (Ly6G, Cat# 561105, 1:500), monocytes/macrophages (CD11b, Cat# 561690, 1:1,000), and dendritic cells (CD11c, Cat# 561044, 1:250). The gating strategy for isolation of these populations was to first exclude dead cells in the population using propidium iodide (PI; Molecular Probes, Eugene, OR, USA). Of the recovered live cells, CD45^+^ immune cells were selected and then sorted into CD11b^+^ monocyte/macrophages/neutrophils and CD11c^+^ dendritic cells. CD11b^+^/c^−^ immune cells were further sorted into CD11b^+^/Ly6G^−^ and Ly6G^+^ to isolate monocyte/macrophages and neutrophils respectively. Viability was 70%–85%, as determined by PI staining. An average of 1.2 × 10^5^ ± 7.2 × 10^3^ live cells were recovered from each blood sample. An average of 3.8 × 10^4^ ± 1.5 × 10^3^ live cells were recovered from each tongue sample. Leukocytes from the spleen were used for compensation controls (i.e., correction of a signal overlap between emission spectra of different fluorochromes used). Data were acquired using a FACSCalibur (BD Biosciences) and analyzed using FlowJo software (Tree Star, San Carlos, CA, USA).

### Fluorescence-Activated Cell Sorting (FACS)

Fluorescence-activated cell sorting (FACS) was used to collect Ly6G^+^ population of immune cells from dissociated tongue tissue treated with HSC-3 cell culture supernatant. Tongue tissue was dissected and dissociated in a manner similar to that used for flow cytometry. To isolate subpopulations, cells were stained with fluorescently conjugated rat anti-mouse mAbs: CD450 (1:400), CD11b (1:1,000), and Ly6G (1:500). PI was used to exclude dead cells. Neutrophils were defined as CD45^+^CD11b^+^Ly6G^+^. Forward and side scatter parameters were used to confirm the size and granularity of the CD45^+^CD11b^+^Ly6G^+^ population. Post-sort purity was >97%. FACS was performed on a three laser, 10 detector FACSAria cell sorter (BD Biosciences). Samples were sorted into RIPA buffer containing protease inhibitor cocktail for protein isolation.

### Enzyme-Linked Immunosorbent Assay

The β-endorphin protein concentration was quantified in tongue tissue from female and male mice with HSC-3 tumors was compared to sham (matrigel alone) mice by enzyme-linked immunosorbent assay (ELISA; MyBioSource, Inc., San Diego, CA, USA). Frozen tissue (20–40 mg) was homogenized in the T-PER Reagent (Pierce Biotechnology, Inc., Rockford, IL, USA) and agitated for an additional 2 h at 4°C. Lysates were centrifuged at 16,000 rpm for 20 min. Cell culture supernatants were removed, aliquoted and protein concentrations were determined using a Bradford Assay (Bio-Rad Laboratories, Inc., Hercules, CA, USA). ELISA was run per the manufacturer’s instructions. The optical density of the standards and samples was read at 450 nm using a Model 680 Microplate Reader (Bio-Rad Laboratories, Inc., Hercules, CA, USA).

### Immunohistochemistry

Animals were euthanized *via* an overdose of inhaled isoflurane and perfused transcardially with 4% paraformaldehyde (PFA, Sigma Aldrich). Tongues were dissected, fixed in 10% neutral buffered formalin, bisected, paraffin-embedded with cut side down, and sectioned at 5 μm thickness through the entire block (about 50 sections). Slide containing the 20th section was selected for staining with anti-Ly6G antibody (Clone 1A8, 1:100, Biolegend, San Diego, CA, USA) by the New York University Langone Medical Center Histopathology Core. Immunoreactions were visualized with diaminobenzidine (DAB) horseradish peroxidase (HRP) substrate kit (Vector Laboratories) and counterstained with Hematoxylin. The sections were photographed using NIS Elements software, a Nikon Eclipse Ti microscope and 2.5× and 60× objectives.

### Statistical Analysis

Analysis of variance (ANOVA) was employed to evaluate the difference between groups regarding sex and treatment. To adjust for multiple comparisons, the *post hoc* Holm-Sidak test statistic was employed. Statistical significance was set at *p* < 0.05. All statistical analyses were performed using Prism (version 8) statistical software (Graphpad Software Inc., La Jolla, CA, USA). Results were presented as mean ± standard error of the mean in box/scatter configuration to show the biological variability.

## Results

### rG-CSF Treatment Increased Oral Cancer-Recruited Ly6G^+^ Neutrophils in the Tongue Microenvironment

**Figure 1:**
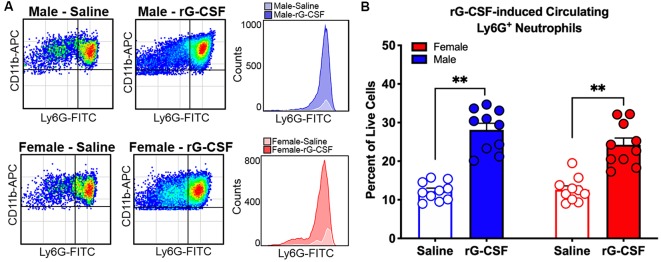
Recombinant granulocyte colony stimulating factor (rG-CSF)-mediated mobilization of neutrophils was quantified using flow cytometry in adult C57Bl/6 mice. **(A)** Representative scatter plots showing CD11b^+^Ly6G^+^ neutrophils in cardiac blood of a naïve male mouse (top) and a female mouse (bottom) 24 h after treatment with saline (left) or rG-CSF (right). Histograms demonstrate the increase in circulating neutrophil after rG-CSF treatment. **(B)** Average Ly6G^+^ neutrophils quantification in cardiac blood from mice (*N* = 10) 24 h following a single treatment of rG-CSF (5 μg/mouse, i.p.) male (blue bars) and female (red bars) mice compared to saline treatment. Unpaired Student’s *t*-test, **P < 0.01.

**Figure 2:**
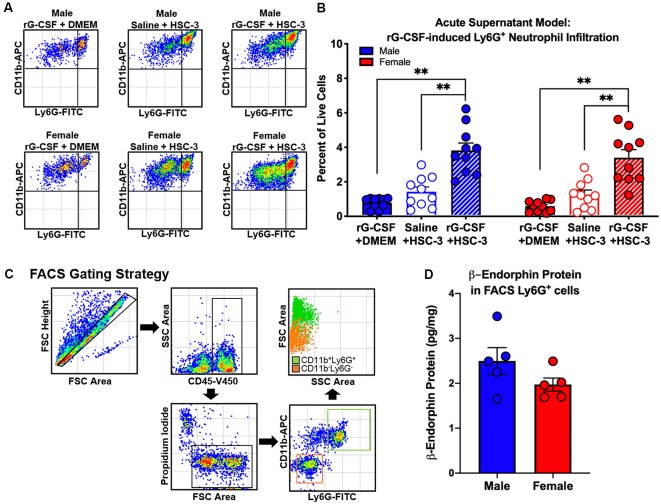
Oral cancer supernatant-induced immune infiltration was measured using the acute supernatant model. **(A)** Representative scatter plots showing CD11b^+^Ly6G^+^ neutrophils from the tongues of an adult C57Bl/6 male (top) and female (bottom) mouse 48 h after either rG-CSF treatment followed by cell culture media (rG-CSF+DMEM, solid bar), saline followed by HSC-3 cell culture supernatant (Saline+HSC-3, open bar), or rG-CSF treatment followed by HSC-3 cell culture supernatant (rG-CSF+HSC-3, striped bar). **(B)** Average Ly6G^+^ neutrophil quantification in the tongues of male (*N* = 10/group, blue bars) and female (*N* = 10/group, red bars) mice after treatment. One-way analysis of variance (ANOVA), **P < 0.01. **(C)** Representative gating strategy used to isolate cancer-activated tongue immune cells by fluorescence-activated cell sorting (FACS). **(D)** Quantification of mean β-endorphin protein in CD11b^+^Ly6G^+^ immune cell subpopulations from HSC-3 supernatant-treated male (blue, *N* = 5) and female mice (red, *N* = 5) relative to total protein. Sorted cells were pooled from two mice for each sample. Unpaired Student’s *t*-test, *P* > 0.05.

To establish a mouse model that permitted assessment of rG-CSF-induced changes in Ly6G^+^ neutrophil infiltration during oral cancer, male and female mice were treated with rG-CSF to increase the percentage of circulating blood Ly6G^+^ neutrophils; the number of circulating Ly6G^+^ neutrophils in the blood was quantified with flow cytometry. Male and female mice treated with 5 μg rG-CSF (i.p.) exhibited significantly more Ly6G^+^ neutrophils in the blood 24 h after injection compared to mice treated with saline (Unpaired *t*-test, male: *t* = 5.368, *P* = 0.001; female: *t* = 2.9178, *P* = 0.022; Figure 1). There was no significant interaction between sex and treatment (two-way ANOVA, *F*_(1,14)_ = 2.1, *P* = 0.163). We previously demonstrated that oral cancer-secreted mediators recruit neutrophils to the tongue cancer microenvironment (Scheff et al., 2017). We sought to determine whether rG-CSF treatment could amplify this effect. Twenty-four hours after rG-CSF treatment, male and female mice received HSC-3 culture supernatant (50 μl) or cell culture media (DMEM) injected into the tongue. The number of infiltrating Ly6G^+^ neutrophils in the tongue was quantified 12 h after supernatant injection (Figure 2A). Administration of rG-CSF treatment prior to HSC-3 supernatant injection (rG-CSF+HSC-3) produced a significant increase in Ly6G^+^ neutrophil recruitment compared to rG-CSF paired with cell culture media (rG-CSF+DMEM) in both male and female mice (one-way ANOVA, male: *P* = 0.001, female: *P* = 0.002; Figure 2B). We also found a significant increase in Ly6G^+^ neutrophils in male and female mice treated with rG-CSF+HSC-3 compared to mice that received saline paired with HSC-3 supernatant (saline+HSC-3; one-way ANOVA, male: *P* = 0.005, female: *P* = 0.014; Figure 2B). There was no significant interaction between sex and treatment (two-way ANOVA, *F*_(2,24)_ = 0.294, *P* = 0.748). To determine if cancer-recruited neutrophils contain opioid protein, CD45^+^CD11b^+^Ly6G^+^ cells were isolated after rG-CSF+HSC-3 supernatant treatment from mouse tongues using FACS (Figure 2C). An average of 124,125 ± 10,092 Ly6G^+^ neutrophils were isolated from each tongue. β-endorphin protein was quantified in cancer supernatant-recruited neutrophils isolated from male and female mice (three samples/sex, each sample contained cells isolated from two mice). There was no significant difference in the quantity of β-endorphin protein detected in neutrophils isolated from male (2.50 ± 0.30 pg/mg) and female (1.97 ± 0.15 pg/mg) mice (Student’s *t*-test, *P* = 0.152; Figure 2D).

### rG-CSF Treatment Abolished HSC-3 Supernatant-Induced Nociceptive Behavior *via* an Endogenous Opioid-Dependent Mechanism

**Figure 3:**
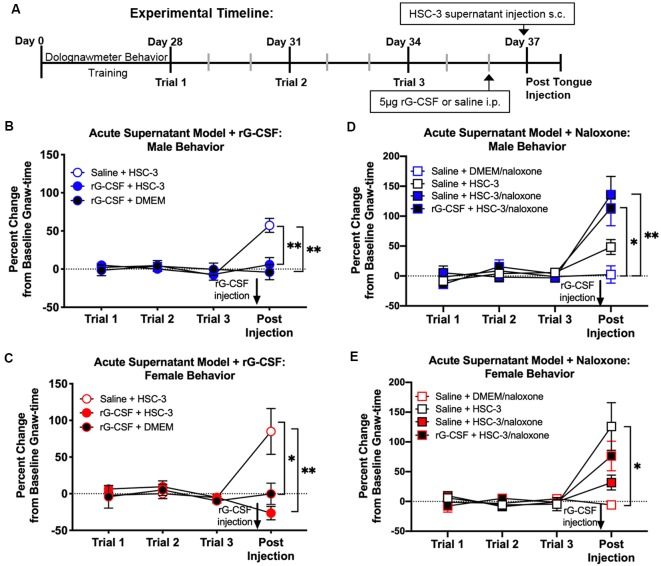
Supernatant-induced nociceptive behavior was measured using the acute supernatant model. **(A)** Schematic of the experimental timeline for an acute supernatant model. Male and female C57Bl/6 mice were trained for 4 weeks in the orofacial pain behavior device and assay (dolognawmeter) or until a steady baseline gnaw-time was reached. Three additional baseline trials were completed and mice underwent a single intraperitoneal injection (i.p.) of saline or 5 μg rG-CSF. Twenty-four hours after treatment, mice received either subcutaneous (s.c.) DMEM (rG-CSF+DMEM) or HSC-3 culture supernatant (saline+HSC-3, rG-CSF+HSC-3, *N* = 8/sex) into the tongue (50 μl) followed by assessment in the dolognawmeter (Post-Injection). A separate group of mice received either s.c. DMEM or HSC-3 cell culture supernatant co-injected with opioid receptor antagonist naloxone (saline+DMEM/naloxone, saline+HSC-3/naloxone, rG-CSF+HSC-3/naloxone, *N* = 5/sex) into the tongue (50 μl) followed by assessment in the dolognawmeter (Post-Injection). Orofacial nociceptive behavior data were analyzed as a percent change from the baseline gnaw-time prior to treatment. Supernatant-induced change in orofacial nociceptive behavior was measured in male **(B,D)** and female **(C,E)** mice and analyzed individually. Two-way ANOVA, *P < 0.05, **P < 0.01 by Holm-Sidak *post hoc* comparisons.

An acute supernatant model was used to quantify oral cancer-induced nociceptive behavior in the absence of tumor burden and illness associated with carcinogenesis (Scheff et al., 2017). Baseline gnaw-times were established using the dolognawmeter. Mice received rG-CSF treatment followed by tongue injection of cell culture media (DMEM) or supernatant (Figure 3A). Nociceptive behavior of mice was assessed 1 h after cell supernatant injection. We found was no difference in gnaw-time between groups prior to treatment; therefore, data were analyzed as a percent change from baseline using two-way ANOVA. There was a significant interaction between time and treatment in both male and female mice (two-way ANOVA, male: *F*_(6,42)_ = 5.025, *P* = 0.005, female: *F*_(6,45)_ = 2.949, *P* = 0.016). HSC-3 supernatant injection yielded an increase in gnaw-time compared to baseline gnaw-time in both male (57.8 ± 9.1%) and female (82.5 ± 18.9%) mice (Holm-Sidak *post hoc*, males: = 0.034, females: *P* = 0.001; Figures 3B,C). Administration of rG-CSF 24 h prior to DMEM (rG-CSF+DMEM) had no effect on gnaw-time compared to baseline gnaw-time in both male (−4.01 ± 9.9%) and female (−0.18 ± 13.2%) mice (males: *P* = 0.998, females: *P* = 0.998; Figures 3B,C). Administration of rG-CSF followed by HSC-3 injection (rG-CSF+HSC-3) significantly limited the HSC-3 supernatant-induced increase in gnaw-time; there was a significant decrease in gnaw-time after supernatant injection compared to saline+HSC-3 treatment in both male and female mice (males: *P* = 0.0002, females: *P* = 0.021; Figures 3B,C).

Peripheral opioid receptor antagonist (naloxone methiodide, 500 μg/kg) revealed an endogenous analgesic mechanism in male and female mice treated with rG-CSF. There was a significant interaction between time and treatment in both male and female mice (two-way ANOVA, male: *F*_(9,66)_ = 4.575, *P* = 0.0001; female: *F*_(9,66)_ = 3.347, *P* = 0.0019). In the absence of rG-CSF, naloxone co-injected with HSC-3 supernatant (saline+HSC-3/naloxone) evoked a significant increase in gnaw-time compared to saline+HSC-3 in male mice (*P* = 0.007) but not in female mice (*P* = 0.052; Figure 3D). After rG-CSF treatment, naloxone co-injected with HSC-3 supernatant (rG-CSF+HSC-3/naloxone) resulted in significantly longer gnaw-times in male mice compared to saline+naloxone (male: *P* = 0.035; Figure 3D). There was no significant difference between rG-CSF+HSC-3/naloxone and saline+HSC-3/naloxone treated male mice (*P* = 0.950, Figure 3D). Five female mice had significantly longer gnaw-time in response to saline+HSC-3 supernatant (*P* = 0.012) when compared to five female mice injected with DMEM/naloxone (Figure 3E). Co-injection with naloxone and HSC-3 supernatant was not significantly different from DMEM/naloxone injection in female mice treated with saline (*P* = 0.998) or rG-CSF (*P* = 0.998; Figure 3E).

### Chronic rG-CSF Treatment Decreased Oral Cancer-Induced Nociception in Female Mice Only

**Figure 4:**
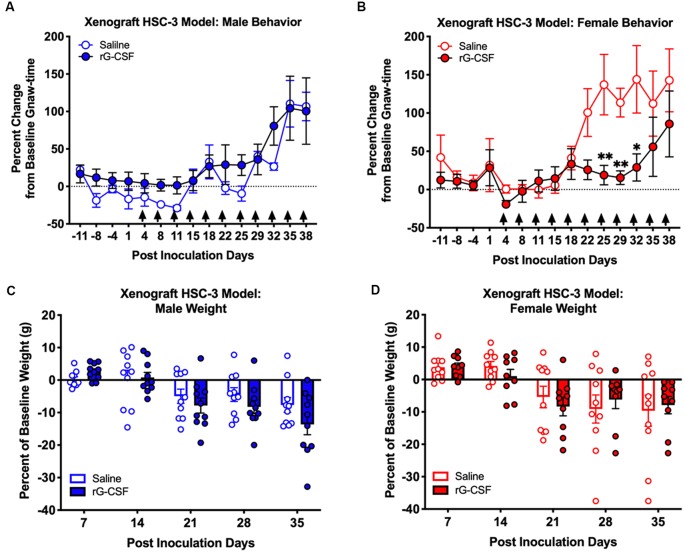
HSC-3 xenograft-induced change in orofacial nociceptive behavior was measured in adult nude male **(A)** and female **(B)** mice receiving saline (*N* = 10, open circles) or rG-CSF treatment (2.5 μg/mouse, *N* = 10, closed circles) 24 h prior to each dolognawmeter measurement, as indicated by the black arrows. Sexes were independently analyzed. Two-way ANOVA, *P < 0.05, **P < 0.01. HSC-3 xenograft-induced weight loss was measured in male **(C)** and female **(D)** mice receiving saline (*N* = 10, open bars) or rG-CSF (2.5 μg/mouse, *N* = 10, closed bars) each week.

We previously demonstrated that Ly6G^+^ neutrophils in the 4NQO-induced oral cancer microenvironment generate endogenous anti-nociception in male mice (Scheff et al., 2018). Neutrophils are present in the cancer microenvironment in the human xenograft HSC-3 mouse model of oral cancer (Ye et al., 2018). Using the HSC-3 xenograft mouse model, mice were treated with 2.5 μg rG-CSF 24 h prior to each behavior assessment beginning at PID 5. Consistent with our previous finding (Scheff et al., 2018), we found a significant interaction between time and sex in saline-treated mice (two-way ANOVA, *F*_(14,224)_ = 2.664, *P* = 0.0013). Holm-Sidak *post hoc* analyses found that tumor-bearing female mice treated with saline had significantly longer gnaw times on PID 25 (*P* = 0.0239), 29 (*P* = 0.0460) and 32 (*P* = 0.0460) when compared to males. When considering rG-CSF treatment, we found a significant interaction between time, sex, and treatment; rG-CSF treatment significantly reduced oral cancer-induced nociceptive behavior in a sex-dependent manner over time (three-way ANOVA, *F*_(14,462)_ = 2.216, *P* = 0.0067). There was no significant difference in gnaw-time between male mice treated with rG-CSF vs. male mice treated with saline (*P* = 0.865; Figure 4A). However, in tumor-bearing female mice, chronic rG-CSF treatment significantly reduced gnaw-time at PID 25 (*P* = 0.009), PID 29 (*P* = 0.022), and PID 32 (*P* = 0.011) compared to saline treatment (Figure 4B). HSC-3 xenograft tumor resulted in a decrease in body weight over time in both sexes (two-way ANOVA interaction, male: *F*_(5,80)_ = 4.992, *P* = 0.0005; female: *F*_(5,90)_ = 3.658, *P* = 0.005); however, there was no significant difference in cancer-induced loss of body mass between saline and rG-CSF treated groups at any time point (two-way ANOVA treatment, male: *F*_(1,16)_ = 1.388, *P* = 0.256; female: *F*_(1,18)_ = 0.330, *P* = 0.572; Figures 4C,D).

### Chronic rG-CSF Treatment Decreased Oral Cancer-Induced Ongoing Nociception in Female Mice Only

**Figure 5:**
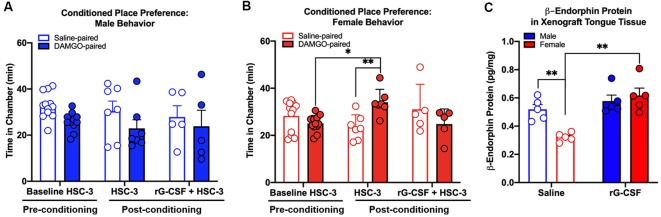
Effect of oral cancer on conditioned place preference (CPP) to DAMGO. Mice were injected with HSC-3 cells into the tongue, habituated on day 21, conditioned with vehicle- and DAMGO (3 μg/kg)-paired chamber for 1 h on days 22–24 and tested for CPP on day 25. Mice received saline (*N* = 7/sex) or rG-CSF (2.5 μg/mouse, *N* = 5/sex) 12 h prior to conditioning. The time spent in each chamber was quantified in tumor-bearing male **(A)** and female **(B)** mice. For all of the CPP experiments, pre-conditioning data (Baseline HSC-3) was analyzed using two-way ANOVA (chambers vs. treatment). Statistical analysis for chamber preference before conditioning revealed no difference in the time spent in chambers between saline and rG-CSF-treated mice (*P* > 0.05), therefore baseline chamber data was pooled for graphical representation. Sexes were independently analyzed. Two-way ANOVA, *P < 0.05, **P < 0.01. **(C)** β-endorphin protein was quantified in homogenized tongue tissue from male (blue bars) and female (red bars) mice with HSC-3 xenograft tumors relative to total protein. Mice treated with either saline (*N* = 5/sex, open bars) or rG-CSF (*N* = 5/sex, closed bars). Two-way ANOVA, **P < 0.01.

In addition to function-related pain, we previously found a significant sex difference in reported intensity of spontaneous pain (Scheff et al., 2018). We used CPP assay (King et al., 2009) to test the hypothesis that rG-CSF treatment can alleviate spontaneous pain secondary to oral cancer in male and female mice. Pre-conditioning (baseline) times did not differ between the vehicle-paired chamber and the drug-paired chamber (*P* = 0.56), therefore data were pooled across groups for graphical representation (Figures 5A,B). There was a sex difference in DAMGO-induced CPP (three-way ANOVA, *F*_(2,48)_ = 6.892, *P* = 0.002). Tumor-bearing male mice did not demonstrate DAMGO-induced CPP regardless of saline (*P* = 0.398) or rG-CSF treatment (*P* = 0.886). However, tumor-bearing female mice treated with saline displayed CPP for the chamber paired with DAMGO; significantly more time was spent in the DAMGO-paired chamber compared with pre-conditioning baseline time (*P* = 0.038) and post-conditioning time in the saline-paired chamber (*P* = 0.008; Figure 5B). rG-CSF treatment prior to conditioning abolished the DAMGO-induced preference in female mice; there was no difference in time spent in the DAMGO-paired chamber compared with the pre-conditioning time (*P* = 0.302; Figure 5B). Pre- and post-conditioning times for the saline (male: *P* = 0.917, female: *P* = 0.996) and DAMGO (male: *P* = 0.889, female: *P* = 0.959) paired chamber did not differ for the sham-treated mice (data not shown).

### Chronic rG-CSF Treatment Increased β-Endorphin Protein in the Oral Cancer Microenvironment in Female Mice Only

To determine whether rG-CSF treatment results in β-endorphin protein in the cancer microenvironment, we measured β-endorphin protein in homogenized tongue tissue from tumor-bearing male and female mice treated with saline or rG-CSF using an ELISA (Figure 5C). There was a significant interaction between sex and treatment (two-way ANOVA, *F*_(1,16)_ = 9.488, *P* = 0.007). Tumor-bearing male mice treated with saline (0.52 ± 0.03 pg/mg) had significantly more β-endorphin protein in the tongue tissue compared to tumor-bearing female mice treated with saline (0.32 ± 0.01 pg/mg, *P* = 0.009). In addition, tumor-bearing female mice treated with rG-CSF (0.62 ± 0.05 pg/mg) had significantly more β-endorphin protein in the tongue tissue compared to tumor-bearing female mice treated with saline (0.32 ± 0.01 pg/mg, *P* = 0.0003, Figure 5C).

### Chronic rG-CSF Treatment Increased Cancer-Recruited Ly6G^+^ Neutrophils in Female Mice Only

**Figure 6:**
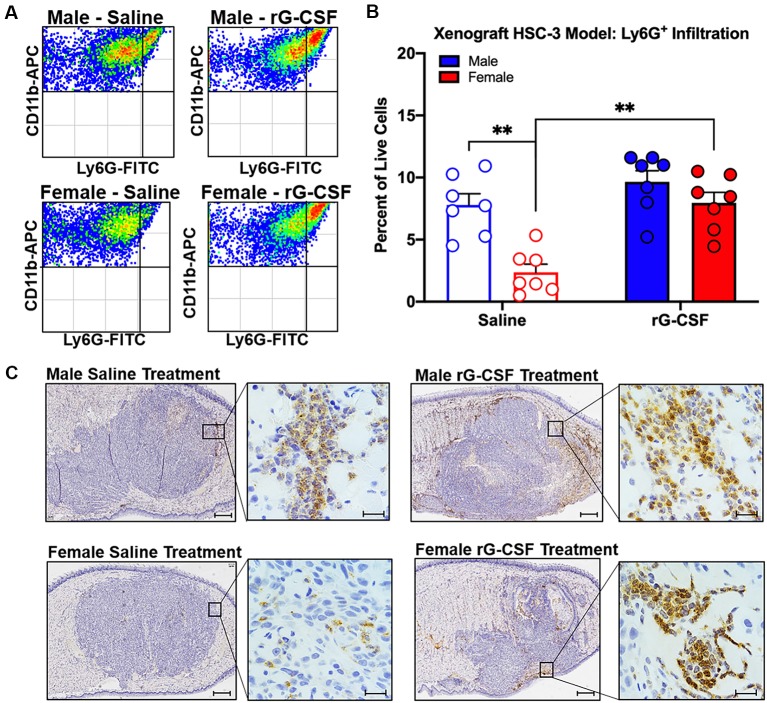
HSC-3 xenograft-induced immune filtration was measured at post-inoculation day (PID) 38. **(A)** Representative scatter plots showing CD11b^+^Ly6G^+^ neutrophils from the tongue of adult male (top plots) and female (bottom plots) nude mice after chronic saline (left side) or rG-CSF (right side) treatment. **(B)** Average Ly6G^+^ neutrophil quantification in the xenograft tongues of male (blue bars) and female (red bars) mice after chronic saline (*N* = 6/sex) or G-CSF (*N* = 6/sex) treatment. Two-way ANOVA, **P < 0.001. **(C)** Immunohistochemical analysis of saline-treated (left) and rG-CSF (right) treated male (top) and female (bottom) mouse tongues. HSC-3 xenograft tumors were stained with Ly6G (1A8) in diaminobenzidine (DAB) and counterstained with hematoxylin. Magnification is 2.5× with 60× insert. Scale bar 500 μm (2.5×) and 20 μm (60×).

**Figure 7:**
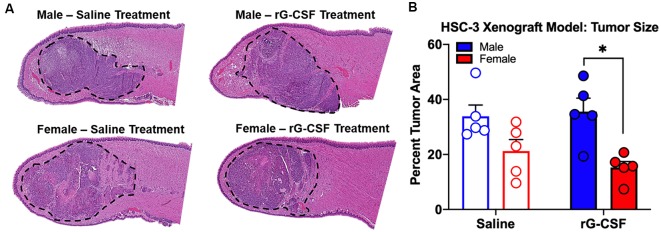
HSC-3 xenograft tumor size was measured at PID 38. **(A)** Representative hematoxylin and eosin-stained sagittal tongue sections (100 μm from center) from male (top) and female (bottom) mice treated with either saline or rG-CSF. HSC-3 tumor is outlined in black dash line. **(B)** Tumor area relative to total tongue area after treatment with either saline (*N* = 5/sex) or rG-CSF (*N* = 5/sex) in male and female mice. Two-way ANOVA, *P < 0.05.

Chronic rG-CSF treatment increased oral cancer-recruited Ly6G^+^ neutrophils in female but not male mice with HSC-3 tumors (two-way ANOVA, *F*_(1,23)_ = 5.098, *P* = 0.033; Figure 6). At PID 38, quantification of immune cells in the tongue revealed significantly more Ly6G^+^ neutrophil recruitment in HSC-3 tumors in female mice treated with rG-CSF compared to saline treatment (*P* = 0.0004). Tumor-bearing male mice treated with saline exhibited significantly more infiltrating Ly6G^+^ neutrophils compared to tumor-bearing females treated with saline (*P* = 0.0006). There was no effect of rG-CSF treatment on HSC-3 tumor Ly6G^+^ neutrophil recruitment in male mice (*P* = 0.5534). Tumor-bearing male mice exhibited a similar number of Ly6G^+^ neutrophils in the oral cancer microenvironment compared to tumor-bearing female mice treated with rG-CSF (Figure 6B). To confirm the identity of the Ly6G^+^ neutrophils, we performed immunohistochemistry on formalin-fixed tongue tissue containing HSC-3 xenograft tumors. Strong anti-Ly6G immuno-like reactivity using DAB HRP was present on the tumor borders in saline-treated male mice as well as rG-CSF-treated male and female mice. Magnification at 60× was used to confirm neutrophil presence based on multi-lobed nuclear morphology (Figure 6C). There was no significant interaction between rG-CSF treatment and sex regarding HSC-3 tumor size in mice (two-way ANOVA interaction, *F*_(1,14)_ = 0.743, *P* = 0.403; Figure 7). However, there was a significant effect of treatment (two-way ANOVA, *F*_(1,14)_ = 13.55, *P* = 0.003); HSC-3 tumor size was larger in rG-CSF-treated male compared to rG-CSF-treated female mice (Holm-Sidak *t* = 3.21, *P* = 0.0370; Figure 7B).

## Discussion

We and others show that women with oral SCC report more pain than men (Reyes-Gibby et al., 2014; Scheff et al., 2018). Human oral SCC produces and releases G-CSF resulting in neutrophil infiltration (Matsuo et al., 1994; Hayashi et al., 1995; Lee et al., 2013). Wang et al. (2014) show that the neutrophil density is higher in men with tongue SCC compared to women. Our previous investigation recapitulates the sex difference in pain and neutrophil infiltration; neutrophil-mediated anti-nociception inhibits nociceptive behavior during the early stage of 4NQO-induced carcinogenesis in male mice (Scheff et al., 2018). In the current study, we tested whether recruitment of neutrophils could be used to reverse oral SCC-induced nociception in female mice. We demonstrate sex-dependent endogenous anti-nociception *via* rG-CSF mediated neutrophil infiltration for the treatment of oral cancer pain.

We report a mechanism to explain the sex difference in oral cancer pain. Sex differences in endogenous anti-nociception may depend on hormonal regulation. Liu and Gintzler (2013) demonstrated spinal mu opioid receptor-mediated anti-nociception depends on circulating estrogen levels. Mogil et al. (1993) find that estrogen contributes to sex-dependent efficacy of naloxone during swim stress-induced analgesia. However, the molecular targets for estrogen in the peripheral nervous system relevant to opioid anti-nociception in oral cancer are not yet defined. Our previous clinical findings of increased cancer pain in females [mean age = 64.5 ± 14.4 (SD) years; Scheff et al., 2018] are consistent with results by Reyes-Gibby et al. (2014) who show that women with oral cancer report more pain than men across 2,340 subjects [mean age = 59 ± 11.7 (SD) years]; these reports demonstrate that postmenopausal women experience significant oral cancer pain suggesting that that mechanisms beyond the estrous cycle contribute to differences in oral cancer pain in men and women.

The underlying cause of the sex difference in neutrophil infiltration remains unidentified; however, gonadal hormones can regulate neutrophils. Estrogen affects the number of circulating neutrophils and neutrophil lifespan (Bouman et al., 2005). Studies using injury and burn rodent models report that testosterone potentiates, whereas estrogen limits calcium mobilization in neutrophils (Deitch et al., 2006). We did not find a sex difference in the number of neutrophils in the blood following rG-CSF treatment in naïve mice. This finding is in contrast to clinical findings in healthy volunteers; Schoergenhofer et al. (2017) identified more plasma neutrophil histone-complexed DNA in the men compared to women after a single of rG-CSF. Consistent with our previous reports in the 4NQO model (Scheff et al., 2018), there is a sex difference in neutrophil infiltration in saline-treated mice with HSC-3 tumors. We also report a sex difference in the efficacy of rG-CSF treatment to drive Ly6G^+^ neutrophil infiltration into the HSC-3 tumor. Chronic rG-CSF treatment in male mice did not increase neutrophil infiltration compared to saline treatment; however, in tumor-bearing female mice, chronic rG-CSF treatment resulted in Ly6G^+^ neutrophil count similar to that found in saline-treated male mice with HSC-3 tumors. One possibility is that maximum neutrophil infiltration has been reached in tumor-bearing male mice and additional recruitment by way of rG-CSF is not physiological. Orofacial nociceptive behavior in saline-treated male mice and rG-CSF-treated female mice with HSC-3 tumors is similar suggesting comparable neutrophil-mediated endogenous anti-nociception present in the cancer microenvironment.

Clinical and preclinical data support the role of rG-CSF for anti-nociception (Brack et al., 2004a; Chao et al., 2012; Ozkaraman et al., 2017). Administration of rG-CSF increases neutrophil count to treat chemotherapy- or radiation-induced neutropenia in patients (Zeidler et al., 2003). In a chronic constriction injury rat model, neutrophils recruited to the site of inflammation following rG-CSF treatment release opioids and alleviate thermal hyperalgesia and mechanical allodynia (Chao et al., 2012). We find that a single rG-CSF treatment produces a significant decrease in nociceptive behavior in response to oral cancer supernatant in both male and female mice. Local naloxone restored supernatant-evoked nociceptive behavior in the presence of rG-CSF. We infer from this result that infiltrating Ly6G^+^ neutrophils in response to rG-CSF treatment are a source of opioid-mediated anti-nociception. Neutrophils secrete opioids in response to inflammatory mediators (Schafer et al., 1994; Rittner et al., 2006; Iwaszkiewicz et al., 2013; e.g., corticotropin-releasing factor, interleukin-1β, and CXCL8) and sympathetic neurotransmitter, norepinephrine (Binder et al., 2004). We find that tumor-bearing female mice treated with rG-CSF have more β-endorphin protein in the tongue tissue compared to tumor-bearing female mice treated with saline. These findings are consistent with Liou et al. (2011) who reported that rG-CSF administered subcutaneously increased opioid content in the injured nerve up to 14 days after partial sciatic nerve injury. Administration of rG-CSF did not affect nociceptive behavior in naïve animals suggesting that neutrophils do not secrete large amounts of opioids in normal circulation.

There are three limitations to our experimental approach. First, we did not investigate and compare opioid receptor expression in male and female mice during oral carcinogenesis in the peripheral nervous system. Endogenous opioids bind to μ- (MOR), δ- (DOR), and κ-opioid receptor (KOR; Kieffer and Evans, 2009). Sexual dimorphism in opioid receptor density is present in the peripheral nervous system; inflammation induced by cytokines or complete Freud’s adjuvant generated significant upregulation of MOR expression in the trigeminal ganglia of male but not female rats (Zhang et al., 2014). A second limitation in our study is the lack of further characterization of neutrophils induced by rG-CSF treatment. Neutrophils in the tumor microenvironment can have a pro-tumor (N2) phenotype capable of supporting tumor growth (Uribe-Querol and Rosales, 2015); depletion of N2 neutrophils decreased lung tumor growth (Fridlender et al., 2009). We find that male mice treated with rG-CSF have larger tumors compared to female mice treated with rG-CSF, despite comparable Ly6G^+^ neutrophil presence in the tongue. Further classification of the rG-CSF-induced tumor-associated neutrophils is necessary to understand the implications of increased neutrophil presence in the oral cancer microenvironment. The third limitation of our work is we did not assess the possible side effects of rG-CSF treatment. G-CSF receptors are broadly expressed on sensory and sympathetic neurons and may modulate tumor-nerve interactions (Schweizerhof et al., 2009; Lambertini et al., 2014; Dobrenis et al., 2015).

Our experimental findings reveal that rG-CSF treatment could hold promise as a future therapeutic approach to oral cancer pain. Our results demonstrated that rG-CSF treatment increases oral cancer-mediated neutrophil infiltration and attenuates oral cancer nociceptive behavior in mouse models. Our results also corroborate sex differences in oral cancer nociceptive behavior and in neutrophil infiltration in the oral cancer microenvironment. We infer from our results that female patients might benefit more from rG-CSF administration because females have fewer neutrophils infiltrating the cancer microenvironment than males.

## Data Availability

All datasets generated for this study are included in the manuscript.

## Ethics Statement

The animal study was reviewed and approved by New York University Institutional Animal Care and Use Committee.

## Author Contributions

All authors listed contributed substantially to the work. NS designed the research, conducted the experiments, performed data analyses, and wrote the manuscript. RA conducted the experiments, performed data analyses, and wrote the manuscript. IW and SY conducted animal behavior and molecular experiments, respectively and performed data analyses. RK provided technical support and data collection. JD provided animal behavior expertise, technical support and edited the manuscript. BS assisted in research design and writing of the manuscript.

## Conflict of Interest Statement

JD fabricates dolognawmeter assays for profit as the single-member limited liability company Gnatheon Scientific. The remaining authors declare that the research was conducted in the absence of any commercial or financial relationships that could be construed as a potential conflict of interest.
